# Different conceptual constructs for modelling sedentary behaviour and physical activity: the impact on the correlates of behaviour

**DOI:** 10.1186/1756-0500-7-921

**Published:** 2014-12-16

**Authors:** Nana Kwame Anokye, Emmanuel Stamatakis

**Affiliations:** Health Economics Research Group (HERG), Brunel University, Uxbridge, Middlesex, London, UB8 3PH UK; Exercise and Sport Sciences, Faculty of Health Sciences, University of Sydney, Sydney, Autralia; Charles Perkins Centre, University of Sydney, Sydney, Australia; Physical Activity Research Group (UCL-PARG), Department of Epidemiology and Public health, University College London, London, UK

## Abstract

**Background:**

Research on the correlates of physical activity (PA) and sedentary behaviour (SB) to date has used independent prediction equations for each behaviour, without considering that they are both part of the same continuum of movement. This assumption of independence might lead to inaccurate estimates because common underlying latent variables may simultaneously influence the propensity to engage in PA and SB. This study tests empirically the interdependent nature of PA and SB by comparing independent equations (current approach in the literature), and joint estimators (a novel but unexplored approach). Using Health Survey for England 2008 data, accelerometry-accessed PA and SB were separately modelled (using ordinary least squared regressions - OLS) and then jointly (using seemingly unrelated regressions -SUR). We tested for diagonality, specification, and goodness of fit.

**Findings:**

The best fit models were the ones that allowed for interdependence of the two movement-related behaviours (*rho* = −0.156; *p* < 0.001). The SUR showed more favourable properties compared to OLS models; producing lower standard errors and more consistent and efficient coefficients. The efficiency gain was more pronounced in the SB equation (Chi^2^ = 92.75; *p* < 0.001).

**Conclusion:**

Evidence from a large national population-wide accelerometry study suggests that accounting for the interdependent nature of PA and SB in prediction equations leads to more efficient modelling estimates. Further research using different samples is, however, required to fully understand the magnitude of efficiency gains accruable from using the joint estimators.

## Background

Physical activity (PA) is an important health behaviour that is linked to cardiometabolic disease risk including obesity [1]. Sedentary behaviour (SB), any low-energy-expenditure activities (≤1.5 MET) in a sitting or reclining posture (e.g. computer use, watching television, driving a car) [2], has been shown to be linked to health risks, even among people who engage in some PA [3–5]. Research to date has been treating PA and SB as distinct entities, although they are part of the same continuum and the above studies [3–5] highlighted some complex interrelationships between them.

In the literature, prediction equations for PA and SB have largely been guided by a key assumption that individuals engage in PA and SB at two discrete time points and when they choose to do one they don’t think of the other. In other words, the two behaviours are independent of each other. This has led many analysts to consider single-equation models, regressing for instance time spent on PA and SB separately on potential correlates [6]. As researchers don’t always observe variables that may have synergies between the decisions to engage in PA and SB, such as environmental factors (e.g. neighbourhood safety), just accounting for observables to study one behaviour at a time, ignoring the other, may be erroneous. This assumption of independence may be limiting because one could argue that individuals’ time allocation in various activities are optimised by their satisfaction space and available time. People could know *a priori* possible scenarios in which they want to do various activities and gain a defined level of satisfaction. Predicting equations for PA and SB may therefore be interrelated to some extent and a failure to account for such inter-correlation could lead to inefficient estimates because it does not make optimal use of all available information [7]. Our earlier empirical work using population-based accelerometry data show a low-to-moderate correlation (rho = −0.28) between moderate to vigorous PA and SB [8], suggesting that this area can be subjected to further empirical testing.

In the present study, we empirically tested the interdependent nature of PA and SB. We compare two different modelling frameworks, namely independent equations (which is current approach in the literature), and joint estimators (which is a promising and novel, but yet unexplored approach) using objectively-assessed PA and SB.

## Methods

### Data

The data source was the 2008 Health Survey for England (HSE08) which had a special focus on PA and SB. HSE08 was a cross sectional survey that drew a roughly nationally representative sample of people residing in private households in England. The sampling was based on a multi-stage stratified random sampling design that used the postcode address file as a sampling frame. A randomly selected sample of 4,507 adults (16 plus years) wore the accelerometer (Actigraph model GT1M, Pensacola, FL,USA). Respondents were to wear (at the waist) the Actigraph during waking hours for 7 consecutive days. Daily use was considered ‘valid’ if the Actigraph was worn for at least 10 hours. Kinesoft software (3.0.98) was used to analyse the raw accelerometry data to generate standardised measures. Further details on the survey and use of accelerometer in the HSE 2008 can be found elsewhere [9].

Like previously [8], moderate to vigorous physical activity (MVPA) was defined as a minutely count of ≥2020 counts/minute and SB was defined as the number of daily minutes with a minutely accelerometry count of <200 counts/minute. We have previously found that in this dataset using different cut off points for sedentary time (e.g. 100 cpm cut point) makes virtually no difference for analysis related to SB [8].

The explanatory factors we included are variables that are commonly correlated with PA and SB [10, 11]. These included socio-demographic variables (age, income, sex, education, ethnicity, marital status, employment status, access to vehicle), household characteristics (number of children and adults in household), health indicators (having a limiting long-standing illness,), health behaviours (smoking and alcohol drinking status) and season of the year participants were measured. At the area level, included correlates were region of residence (strategic health authourity) and urbanisation.

### Statistical analysis

Two multivariable modelling frameworks were used to estimate the factors that are associated with MVPA and SB. First, following the current empirical approach in the literature, separate OLS models were fitted for MVPA and SB respectively, assuming that MVPA and SB are independent. The estimates from such an approach served as baseline for us to test the joint nature of the two lifestyle choices. The assumption underlying this approach was that the error terms of both equations were not correlated [7]. The second approach fitted a seemingly unrelated regression model (SUR), which jointly and simultaneously estimates equations for MVPA and SB. This estimator accounts for the correlation between both equations. In practice, the seemingly unrelated model is estimated using two joint linear models that indicate an individual’s participation in MVPA(Y_1_) and SB (Y_2_) correspondingly:
12

where X_1_ and X_2_ are regressors of PA and SB respectively. X_1_ ≠ X_2_ and we also require T > K_i_ (where T = total observations; K_i_ = total regressors).


regressors are assumed to be strictly exogenous:


For any given equation the disturbance is homoscedastic and that the errors terms are uncorrelated across observations but correlated across Eqs. (1) and (2). Therefore:


Comparative analysis of the SUR and OLS was conducted with a number of indicators. Using the test for diagonality, Breusch Pagan test, we examined whether SUR compared with the OLS leads to efficiency gain. This test produces a LM statistic that adds the squared correlations between the residual vectors for equations (for MVPA, and SB), with a null hypothesis of diagonality, zero contemporaneous covariance between the disturbances of the two equations [12]. The size of standard errors of estimated parameters of both equations was also compared in relative terms (specified as: (StandardError_OLS_- StandardError_SUR_/StandardError_SUR_)*100)). The Hausman specification test was used to check which estimator produced better consistent and efficient estimates.

The models were estimated with sampling weights that were calculated as the inverse of the probability of being a respondent in a household multiplied by the household weight which accounts for non-responding households [9]. As individuals are nested within households, the models were estimated with cluster (household identifier) to allow the errors associated with individuals residing in a household to correlate with each other.

The PA data was log-transformed using the default logarithmic scale of [u = log10] to improve normality of its distribution. Marginal effects (or elasticity for continuous correlates) were computed to show relative impact of a correlate on MVPA or SB. In the case of equations for MVPA, marginal effects were expressed as exponential values because the associated geometric means (as showed by the marginal effect) arising from log transformed dependent variables has to be converted to the arithmetic mean for comparison with the original data for interpretation. The t-test was used to examine whether missing data occurred completely at random. If not at random, missing values for explanatory variables were treated as separate categories and included in the models in order to avoid biased estimates [13]. The threshold for statistical significance was set at ≤5 % in all analyses. Multiple comparisons were adjusted for using Bonferroni correction. All analyses were undertaken using Stata version 13.

## Results

### Description of sample

Descriptive statistics for the variables are presented in Table 1. A total of 2,289 adults had valid accelerometry data and were included in the analyses. On the average, people spent 28 minutes/day participating in MVPA, and 472 minutes undertaking SB per valid day. The mean age of sample was 52 (SD = 18) years. Most were female (55%), married and living with their partners (55%) and employed (54%). Few were obese (BMI ≥ 30 kg/m^2^) or current smokers (20%), while the large majority were drinkers (89%), defined as drinking alcohol at once or twice a year.

**Table 1:** **Descriptive statistics of variables**

Variables	Observations	Mean(SD)/%*
*Dependent*		
Time spent undertaking moderate to vigorous physical activity per day (mins)	2289	28.2(25.4)
Time spent undertaking sedentary activity per day (mins)	2289	472.1(126.4)
*Explanatory variables*		
Age	2289	51.7(18)
Income (%)		
*>44200*	451	19.7
*<17789*	680	29.7
*>/=17789 and <27317*	389	17
*>/=27317 and <44200*	403	18
*missing*	366	16
Sex		
*Males*	1,030	45
*Females*	1,259	55
Educational qualification		
*No qualification*	601	26.3
*Degree equivalent*	472	20.6
*Higher education below degree*	271	11.8
*‘A’/ ‘0’ level/NVQ*	714	31.2
*Other qualification*	231	10.1
Ethnicity		
*White British*	244	10.7
*Other*	2,045	89.3
Marital status		
*Single*	533	23.3
*Married and living with partner*	1255	54.8
*Other*	501	21.9
Employment status		
*Unemployed*	195	8.5
*Employed*	1244	54.4
*Retired*	693	30.3
*Looking after family*	157	6.9
Health status		
*Limiting illness*	603	26.3
*Non-limiting illness*	506	22.1
*No illness*	1,180	51.6
Smoking status		
*Non-smoker*	1,075	47
*Former smoker*	743	32.5
*Smokers*	465	20.3
*Missing*	6	0.3
Drinking status		
*Almost every day*	324	14.2
*Five or six days a week*	129	5.6
*Three or four days a week*	363	15.9
*Once or twice a week*	603	26.3
*Once or twice a month*	291	12.7
*Once every couple of months*	147	6.4
*Once or twice a year*	184	8
*Not at all in the last 12 months/non-drinkers*	243	10.6
*Missing*	5	0.2
Number of children in household		
*No child*	1932	84.4
*One children*	190	8.3
*Two or more children*	167	7.3
Number of adults in household		
*One adult*	515	22.5
*Two adults*	1427	62.3
*Three or more adults*	347	15.2
Seasons		
*Winter*	589	25.7
*Autumn*	708	30.9
*Summer*	442	19.3
*Spring*	550	24
Region of residence		
*North east*	162	7.1
*North west*	339	14.8
*Yorkshire and the Humber*	248	10.8
*East midlands*	271	11.8
*West midlands*	232	10.1
*East of England*	271	11.8
*London*	190	8.3
*South east coast*	176	7.7
*South central*	133	5.8
*South west*	267	11.7
Urbanisation		
*Urban*	1841	80.4
*Town & fringe*	203	8.9
*Village, hamlet and isolated dwellings*	245	10.7
Access to vehicle		
*Yes*	362	15.81
*No*	1,927	84.19

*Mean (SD) refers to numbers with parenthesis beside them. Otherwise, percentages.

Only three variables (income, smoking status, and drinking status) had missing observations. ‘Income’ had the highest number of missing observations (*n* = 366), whereas ‘drinking status’ had the lowest (*n* = 5). Daily MVPA of respondents with missing values were different from those without (income: 26.28(SD = 1.25) vs 28.61(0.56), p value = 0.107; smoking status: 60.33(SD = 23.41) vs 28.15(0.53), p value = 0.002; drinking status: 76.60(SD = 28.18) vs 28.13(0.53), p value <0.001).

### Comparison of modelling frameworks

Tables 2 and 3 show estimates for the results of SUR and OLS models for MVPA, and SB respectively. The MVPA equation was found to be correlated with that of SB equation (*r* = −0.156; P <0.001), with the Breusch-Pagan test for independence of the residual vectors of both equations suggesting such correlation is not by chance.

**Table 2:** **Estimation results of seemingly unrelated regression model (SUR) and ordinary least square (OLS): physical activity**

Explanatory variables	Physical activity				
	OLS		SUR		% difference in SE (SUR vs. OLS)
	*Coef. (ME)* ^*a*^	*SE* ^*b*^	*Coef. (ME)* ^*a*^	*SE* ^*b*^	
Age	−0.030(−0.470)***	0.002	−0.030(−1.596)***	0.002	0.57
Sex					
*Males* ^*c*^					
*Females*	−0.253(0.776)***	0.046	−0.253(0.759)***	0.046	0.28
Health status					
*Limiting illness* ^*c*^					
*Non-limiting illness*	0.532(1.702)***	0.073	0.531(1.744)***	0.073	0.32
*No illness*	0.492(1.636)***	0.065	0.491(1.704)***	0.065	0.27
Drinking status					
*Almost every day* ^*c*^					
*Five or six days a week*	0.363(1.438)**	0.112	0.363(1.415)**	0.112	0.41
*Three or four days a week*	0.106(1.112)	0.090	0.107(1.101)	0.090	0.34
*Once or twice a week*	0.076(1.079)	0.077	0.077(1.028)	0.077	0.32
*Once or twice a month*	−0.013(0.987)	0.089	−0.012(0.920)	0.088	0.39
*Once every couple of*					
*months*	−0.102(0.903)	0.108	−0.101(0.848)	0.108	0.30
*Once or twice a year*	−0.233(0.792)	0.109	−0.230(0.763)	0.109	0.39
*Not at all in the last 12 months/non-drinkers*	−0.257(0.773)	0.109	−0.253(0.762)	0.108	0.40
Access to vehicle					
*No* ^*c*^					
*Yes*	−0.228(0.796)**	0.068	−0.210 (0.865)**	0.067	1.94
*Constant*	4.187***	0.127	4.172***	0.125	1.37
*Observations*	2268		2268		

^a^Coefficient (Marginal Effects/Elasticity); ^b^Standard Error; ^c^Omitted category *Exponentiated ME > 1(negative effect), exponentiated ME <1(negative effect); Significance level of 1% (***), 5% (**); Hausman specification test (null hypothesis: difference in coefficients in two models is not systematic): Chi2 (26) = 0.10; *p* = 1.000.

**Table 3:** **Estimation results of seemingly unrelated regression model (SUR) and ordinary least square (OLS): sedentary behaviour**

Explanatory variables	Sedentary behaviour				
	OLS		SUR		% difference in SE (SUR vs. OLS)
	*Coef. (ME)* ^*a*^	*SE* ^*b*^	*Coef. (ME)* ^*a*^	*SE* ^*b*^	
Age	2.150 (0.219)***	0.325	2.239 (0.253)***	0.315	3.25
Sex					
*Males* ^*c*^					
*Females*	−30.439(−30.439)***	6.226	−32.612(−30.166)***	6.105	1.98
Educational qualification					
*No qualification* ^*c*^					
*Degree equivalent*	52.937(52.937)***	10.924	53.544(47.168)***	10.472	4.29
*Higher education below degree*	30.421(30.421)	10.656	31.813(23. 752)	10.206	4.40
*‘A’/‘0’ level/NVQ*	11.724(11.724)	8.954	14.274(15. 429)	8.553	4.69
*Other qualification*	3.658(3.658)	12.213	3.135(2.859)	12.006	1.72
Health status					
*Limiting illness* ^*c*^					
*Non-limiting illness*	−17.590(−17.590)	7.960	−14.058(−14.351)	7.594	4.82
*No illness*	−25.601(−25.601)***	6.774	−26.444(−22.749)***	6.710	0.96
Smoking status					
*Non-smoker* ^*c*^					
*Former smoker*	6.512(6.512)	6.851	2.999(0.218)	6.589	3.97
*Smokers*	−24.422(−24.422)**	9.383	−29.638(−25.870)**	9.050	3.68
*Constant*	393.172***	27.129	393.525***	26.778	1.31
*Observations*	2289		2268		

^a^Coefficient (Marginal Effects/Elasticity); ^b^Standard Error; ^c^Omitted category; Significance level of 1% (***), 5% (**); Hausman specification test (null hypothesis: difference in coefficients in two models is not systematic): Chi2 (44) = 92.75; *p* = 0.000.

Second, further evidence of efficiency gains via the SUR can be ascertained by comparing standard errors. The magnitude of reduction in standard errors achieved via SUR was found to be relatively more for the estimated parameters of SB equation compared to the MVPA one (see sixth columns of Tables 2 and 3).

Third, the Hausman specification test suggested that for SB, the parameters of the SUR model were systematically different from those of the OLS (Chi^2^ = 92.75; P <0.001) and produced better consistent and efficient estimates. Both models, however, yielded similar coefficients in the case of MVPA. Table 2 shows both SUR and OLS indicate that older individuals, females, and individuals with access to vehicles were associated with lower levels of MVPA. Conversely, individuals who had non-limiting illness (or no illness) undertook more PA per day. Compared with individuals who drank alcohol almost every day, people who drank on 5 or 6 days/week spent more time undertaking MVPA (ME = 1.415 to 1.438). The correlates of SB differed from that of MVPA in terms of type of correlates as well as direction of correlation (when same correlates were found across both behaviours; except for gender). As shown on Table 3, SB was positively correlated with age, and educational qualification. Females, individuals in better health, and smokers spent less SB time.

## Discussion

This is the first study, to our knowledge, to conduct an empirical test as to which of the two conceptual constructs (i.e. whether PA and SB are jointly determined or independent) is likely to be more efficient modelling framework. A search of SCOPUS (largest bibliographic database) and PUBMED, conducted in May 2014, located no applications of joint estimators for PA and SB, although two previous studies have applied SUR to PA and sport or diet [14, 15]. The findings from the analysis in this study showed a contemporaneous correlation between the errors terms of equations for SB and PA, suggesting that the two movement behaviours are interdependent and hence the utility of the SUR particularly for SB (albeit small efficiency gains).

In addition, the SUR provided relatively less uncertain coefficients than the OLS estimator, especially in the case of SB. Therefore, joint estimators were found in this study to be a more efficient modelling framework than the current approach in the literature (single estimators). Notably, if there was no evidence of a contemporaneous correlation between the SB and MVPA equations, the SUR would have been equivalent to equation by equation via OLS. Nonetheless, the SUR would have still been worthwhile because it has an attractive feature of allowing restrictions to be imposed and appropriate tests conducted across parameters in the different equations. For example, joint significance tests for variables that are common to both equations could be undertaken.

Our analysis is not free from an important limitation though; but we judge that the implications of this limitation for our conclusions if any are only minimal. For example, our analysis had unequal observations on which the MVPA and SB equations were estimated (the former had 20 less observations). Noted by McDowell [16], fitting a SUR on equations that have varying number of observations could lead to loss of information because observations that are unavailable for both equations are discarded, potentially leading to more uncertain estimates. In our case, because the excluded observations were not systematically different from the included ones and fitting the equations on the full set of imputed observations resulted in similar findings, it is unlikely that the current conclusions would change if actual information were available on those 20 observations.

Notwithstanding the limitation, the findings from this study do offer an important consideration for future research on SB and PA. The key message here is that to achieve more efficient and tighter estimates, analysis of the correlates of PA and SB in particular ought to use simultaneous joint equations that account for the intercorrelation between PA and SB. Further research using different samples is, however, required to fully understand the magnitude of efficiency gains accruable from using the joint estimators. Only by examining this, will we be in a position to accurately determine the importance of joint estimators in this area.

## Conclusion

This is the first study examining the efficiency gains accruable to joint estimators (e.g. SUR based analysis) into the associated factors of PA and SB. However, it would be important to replicate these results in other datasets in order to provide firmer conclusions on the most appropriate modelling framework for analysing PA and SB.

## Abbreviations

PA: Physical activity
SB: Sedentary behaviour
SUR: Seemingly unrelated regression
OLS: Ordinary least squares.
